# Enkephalins and Pain Modulation: Mechanisms of Action and Therapeutic Perspectives

**DOI:** 10.3390/biom14080926

**Published:** 2024-07-30

**Authors:** Mario García-Domínguez

**Affiliations:** Faculty of Education and Psychology, Universidad Francisco de Vitoria, 28223 Pozuelo de Alarcón, Spain; mario.gdominguez@ufv.es

**Keywords:** opioid peptide, analgesia, enkephalin, pain, disease, pathology

## Abstract

Enkephalins, a subclass of endogenous opioid peptides, play a pivotal role in pain modulation. Enkephalins primarily exert their effects through opioid receptors located widely throughout both the central and peripheral nervous systems. This review will explore the mechanisms by which enkephalins produce analgesia, emotional regulation, neuroprotection, and other physiological effects. Furthermore, this review will analyze the involvement of enkephalins in the modulation of different pathologies characterized by severe pain. Understanding the complex role of enkephalins in pain processing provides valuable insight into potential therapeutic strategies for managing pain disorders.

## 1. Introduction

The International Association for the Study of Pain (IASP, 2020) has defined *pain* as an “unpleasant sensory and emotional experience associated with, or resembling that associated with, actual or potential tissue damage” [1,2]. This definition illustrates that pain is a complex event that includes not only a sensory experience but also integrates emotional and cognitive aspects [1,2].

Pain acts as a crucial diagnostic indicator, frequently indicating health problems that require medical care [3]. Pain can be categorized into various categories based on different characteristics, such as origin or duration. *Acute pain* typically occurs due to injury or illness and is transient. However, *chronic pain* persists over an extended period (at least three months), often beyond the expected time for tissue healing, and may be linked to underlying pathological conditions [4,5]. When pain becomes chronic, physical, emotional, and social challenges arise [6].

The epidemiology of pain provides valuable insights into its global prevalence, demographic variations, and public health implications [7,8]. Chronic pain affects a significant portion of the global population, with approximately 30% of individuals experiencing it. The prevalence of chronic pain increases with age [9,10,11,12]. Pathologies like migraines, low back pain or rheumatoid arthritis are the most prevalent causes of chronic pain [10,11]. Women tend to experience chronic pain more frequently than men [10,11,12].

Variations in pain prevalence across countries are influenced by several factors, such as healthcare accessibility and socioeconomic circumstances [7,10]. Developed countries frequently show higher prevalence rates due to advanced diagnostic resources and longer life expectancies, whereas pain is commonly underreported in developing countries [10,11]. The economic impact of chronic pain is substantial, involving direct medical costs and indirect costs such as decreased employee productivity [8]. Chronic pain is a leading contributor to disability worldwide [13], underscoring the importance of effective pain management strategies and policies to improve the healthcare systems.

Pain management requires a deep understanding of its underlying causes. Clinicians must have the proficiency and resources to accurately evaluate pain, considering several factors like its severity, duration, and impact on daily activities [5]. Implementing a patient-centered approach is crucial in pain management, ensuring that treatment strategies are customized to the individual circumstances [14]. This fact implies the implementation of a multidisciplinary strategy, integrating pharmacological treatments, and psychophysical therapies [14].

However, many vertebrates (including mice, rats, and humans) have physiological mechanisms that significantly reduce nociception and, as a result, the intensity of pain [15,16]. The primary mechanism for pain control involves the activation of the *endogenous opioid system*, where *opioid peptides* bind to receptors located predominantly in the nervous system, leading to analgesic effects [16,17,18,19,20].

In 1975, John Hughes reported the first evidence of endogenous opioids in brain extracts through the observation of their capacity to inhibit acetylcholine release from nerves in the guinea pig ileum [21]. Furthermore, he indicated that this inhibition was completely blocked by the action of the non-selective opioid receptor antagonist naloxone. The compound that he isolated was termed *enkephalin* (ENK), a subclass of opioid peptide [21].

ENKs are essential in pain regulation, acting as neurotransmitters that modulate pain signals in the central (CNS) and the peripheral nervous system (PNS) [22,23,24,25]. Through binding to opioid receptors, these peptides inhibit the release of neurotransmitters that contribute to the perception of pain, thereby providing a natural mechanism for pain relief [22,26,27]. Diverse research reviewed that manipulating enkephalinergic pathways could provide new therapeutic approaches for addressing both acute and chronic pain [28]. This circumstance underscores the significance of continuous research into these mechanisms to develop more effective treatments. Moreover, ENKs induce other effects such as emotional regulation [29], motor control [30], and the regulation of appetite and feeding behavior [31].

This review will cover essential aspects of ENKs in pain management, including their synthesis and degradation, distribution, and their role in several physiopathological processes. Lastly, the effectiveness of different drugs that enhance the effects evoked by ENKs in painful diseases will be analyzed.

## 2. Molecular Biology of the Enkephalins and Their Receptors

Currently, two primary ENKs are recognized, distinguished by their carboxy-terminal amino acid [32,33]: *met-enkephalin* (Met-ENK; Tyr-Gly-Gly-Phe-Met) and *leu-enkephalin* (Leu-ENK; Tyr-Gly-Gly-Phe-Leu). Moreover, Met-ENK has several variants [34]: Met-ENK-Arg^6^-Phe^7^ (MEAP) and Met-ENK-Arg^6^-Gly^7^-Leu^8^ (MEAGL).

ENKs are synthesized across numerous CNS and PNS regions, immune cells, and adrenal glands [23,24,25,35,36,37] through the transcription of the proenkephalin gene (*Penk*) and the subsequent enzymatic cleavage of the proenkephalin A protein (pENK; 243 αα) [34]. However, ENKs can be produced from *β-endorphin* (β-END) and/or *dynorphin* (DYN) through posttranslational processing due to the sequence homology among these peptides [38].

**Figure 1:**
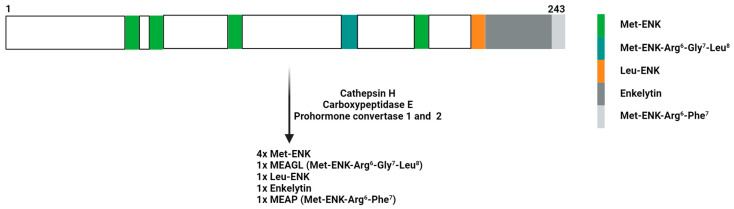
General schedule of the pENK protein. Proteolytic cleavage of the pENK protein results in four copies of Met-ENK, and one copy each of Leu-ENK, MEAP (Met-ENK-Arg^6^-Phe^7^), MEAGL (Met-ENK-Arg^6^-Gly^7^-Leu^8^), and enkelytin.

The maturation of pENK into functional opioid peptides (four copies of Met-ENK and one copy of Leu-ENK) requires the synchronized action of numerous peptidases (Figure 1), including prohormone convertase 1 and 2, carboxypeptidase E, and cathepsin H [39,40,41]. Once released, ENKs are processed by three peptidases (aminopeptidase N, neprilysin, and angiotensin-converting enzyme or ACE) to regulate the half-life of these peptides [42].

Met-ENK and Leu-ENK exhibit the greatest affinity for δ-opioid receptors (DOPr), although they can also bind µ-opioid receptors (MOPr) [43,44]. DOPr is a protein that belongs to the family termed G-protein coupled receptors (GPCR; 372 αα), characterized by their seven membrane-spanning motifs [45,46]. The core sequence of DOPr (encoded by the *Oprd1* gene) shows high homology (90%) across many mammalian species such as mouse, rat, and human [47,48,49]. The *Oprd1* gene is divided into three exons and no alternative splicing had been documented, but recent findings in mice and humans suggest alternative transcriptional processes, indicating extensive variations in DOPr splicing [50,51]. DOPr are distributed throughout the nervous system and other organs, such as the heart, lungs, or gut [52,53].

**Figure 2:**
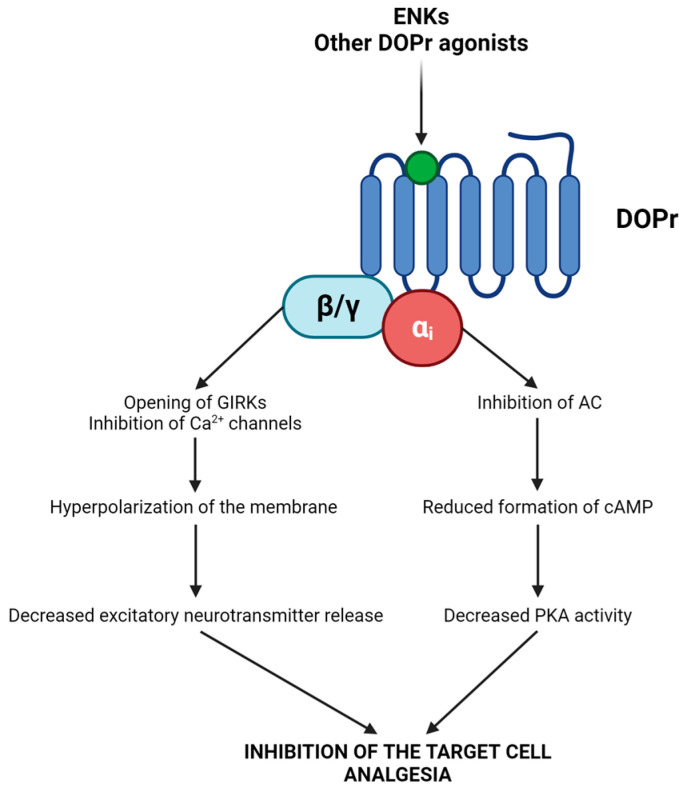
Actions evoked by ENKs and other DOPr agonists. The effect is the inhibition of the target cells and/or the induction of analgesia.

Signal transduction initiates when ENKs bind to DOPr, prompting the dissociation of the Gα_i_ and Gβγ subunits (Figure 2) [54,55,56]. The Gα_i_ subunit inhibits adenylyl cyclase (AC), resulting in decreased cAMP formation and subsequently reduced protein kinase A (PKA) activity [54,55,56]. Gβγ leads to the opening of G-protein-gated inwardly rectifying K^+^ channels (GIRKs; inducing membrane hyperpolarization) and decreases Ca^2+^ influx by binding to numerous types of Ca^2+^ channels (causing decreased neurotransmitter release) [54,55,56]. The consequence is the inhibition of the target cells and/or the induction of analgesia [52,53,54,55,56].

Epigenetics is an emerging area of scientific research that examines how environmental factors influence gene expression without modifications in the DNA sequence [57]. Several studies have confirmed that the *Oprd1* and *Penk* genes are regulated by epigenetic mechanisms [58,59,60,61,62,63].

In studies involving neuroblastoma cells, *Oprd1* is transcriptionally active in undifferentiated cells but becomes inactive during neuronal differentiation [58]. This variation results from differences in promoter methylation [58]; the promoter is hypomethylated in undifferentiated cells and hypermethylated in differentiated cells [58]. Without methylation, the *Oprd1* promoter in neuroblastoma cells remains accessible. Conversely, in neural cells where the *Oprd1* promoter is methylated, accessibility is significantly decreased [58]. This fact implies that promoter methylation reduces the accessibility of RNA polymerase II [58]. Beyond the effect of cellular differentiation on *Penk* promoter methylation, diet is also a critical factor. Specifically, high-fat diets are associated with lower levels of *Oprd1* gene methylation in the human brain [59].

Conversely, *Penk* expression is specific to cell types and tissue compartments [60] and can be influenced by environmental factors such as smoking [61] and sunlight [62]. Analysis of the DNA sequence has demonstrated that certain CpG sites are methylated in a tissue-specific manner, highlighting the significant role of promoter methylation in the regulation of *Penk* expression [63].

## 3. Distribution and Functions of Enkephalins

### 3.1. Central Nervous System (CNS)

**Figure 3:**
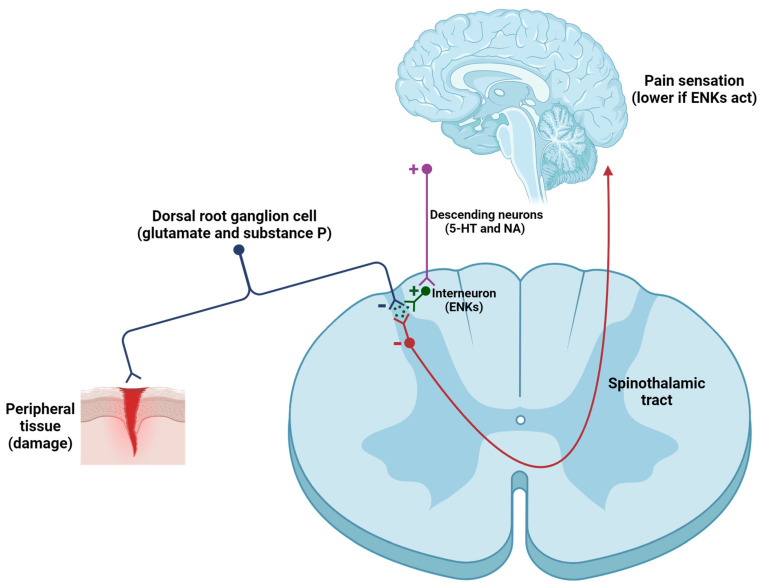
Modulation of the nociceptive pathway induced by the release of ENKs in the spinal cord. When tissue damage occurs, primary afferent neurons are activated and release neurotransmitters and neuropeptides (mainly glutamate, substance P, and CGRP) onto second-order neurons. These neurons transmit information to higher centers, leading to the sensation of pain. However, activation of the descending pain pathway, involving serotonergic (5-HT) and noradrenergic (NA) neurons, results in the activation of spinal interneurons that contain ENKs (among other substances). ENKs inhibit first-order and second-order neurons (both contain DOPr). The result is a significant reduction in inputs to the cortex, resulting in a smaller sensation of pain.

ENKs have been shown to be extremely widely distributed throughout the CNS of vertebrates, including humans [64]. In rats, the prosencephalic structures where ENK fibers were discovered are cortex, lymbic system, striatum, hippocampus, thalamus, and hypothalamus [65,66,67,68,69,70]. Regions in the midbrain include periaquaductal grey area and reticular formation [71,72] and regions in the hindbrain comprise locus ceruleus or nucleus tractus solitarii [73,74]. In the spinal cord of rats, ENK fibers were revealed in the dorsal horn, particularly in the superficial layers, known as laminae I and II [75,76]. In humans, ENK fibers were located in nucleus accumbens, caudate nucleus, globus pallidus, hypothalamus, substantia nigra, locus coeruleus, and spinal cord (mainly the lamina II) [77,78,79,80,81,82,83]. This distribution of ENKs across the CNS confer them a wide range of physiological functions:

1. Pain modulation. One of the primary mechanisms by which ENKs modulate pain involves the inhibition of the transmission of nociceptive signals in the dorsal horn. ENKs, released by spinal interneurons, exert postsynaptic effects within the CNS (Figure 3), because they suppress the activity of ascending neurons (activated by excitatory neurotransmitters -such as glutamate, substance P, and calcitonin gene-related peptide or CGRP, secreted by primary afferent neurons) [84,85,86,87,88,89]. Furthermore, ENKs indirectly activate serotonergic neurons in the raphe nucleus and noradrenergic neurons in the locus coeruleus, thereby promoting the activation of the descending pathway, which in turn regulates pain intensity [85]. To summarize, ENKs can alter the function of GABAergic, noradrenergic, serotoninergic, and glutamatergic neurons, thereby affecting the equilibrium of excitatory and inhibitory neurotransmission within pain pathways [86].

2. Emotional control. The mood-modulating effects evoked by ENKs are largely attributed to the alleviation of pain sensation. These effects are similar to those observed following the administration of exogenous opioids, like morphine or fentanyl [17]. Moreover, ENKs are involved in the stress response by modulating the hypothalamic-pituitary-adrenal (HPA) axis, thereby affecting cortisol secretion, the main stress hormone [90].

A critical region where ENKs exert their biological effects is the paraventricular nucleus of the hypothalamus, where corticotropin-releasing factor (CRF) neurons are located. Several studies have demonstrated that ENKs inhibit the secretion of CRF [33,90,91]. This suppression prevents the overactivation of the HPA axis, thereby regulating the acute and chronic stress response [33,90,91].

Additionally, ENKs play an essential role in influencing the reward pathways, which are vital for reinforcing behaviors for survival, such as feeding and reproduction [22,92,93]. The most crucial reward pathway in the brain is the mesolimbic pathway, which includes the nucleus accumbens and the ventral tegmental area, centers rich in DOPr and MOPr [94,95]. Activation of these receptors by ENKs promotes the biosynthesis and secretion of dopamine, a neurotransmitter linked to pleasure and reward, promoting feelings of satisfaction and positive reinforcement [92]. This mechanism not only supports natural reward processes, but also plays a role in modulating addictive behaviors [94,95]. Through their influence on the reward circuitry, ENKs can influence the susceptibility to substance addiction, emphasizing their potential in addiction therapy [94,95].

Finally, ENKs enhance the pleasure derived from food intake [96,97]. The release of ENKs, triggered by consumption, promotes feelings of satisfaction and pleasure, thereby influencing food choices [96,97].

3. Neuroprotective effects. The role of ENKs in neuroprotection is an emerging field of interest. Several studies have demonstrated that ENKs can exert neuroprotective effects by reducing toxicity, particularly in those cases where excessive glutamate release leads to neuronal damage [98,99]. Another function induced by ENKs is the attenuation of oxidative stress in the CNS following pathological disorders, as seen in stroke [99], Alzheimer’s disease [100], or Parkinson’s disease [101,102].

### 3.2. Peripheral Nervous System (PNS)

In addition to its widespread distribution throughout the CNS, ENKs are distributed in peripheral neuronal and paraneuronal elements. Among numerous regions of the PNS where peptide localization has been determined, the enteric nervous system (ENS) is distinguished by its abundance of ENK neurons, making enteric ganglia particularly intriguing [103,104,105]. ENK neurons have been identified in the gut walls of mammals, including mouse, rat, guinea pig, and human [106,107,108]. Specifically, ENKs are found within the neuronal cells of Meissner’s and Auerbach’s plexuses [103,104,105].

Additionally, ENK neurons have been found in various sympathetic ganglia (cervical ganglion, inferior and superior mesenteric ganglia) in guinea pig, mouse, rat, and human [109,110]. ENK neurons were also detected in the adrenal medulla [111]. Finally, the presence of ENKs in the parasympathetic nervous system has been determined [112]. For instance, the vagus nerve has been demonstrated to contain fibers with diverse neuropeptides, including ENKs [112].

The distribution of ENKs within the PNS provides them with a diverse range of functions:

1. Control of inflammation and pain modulation. Inflammation is the physiological response to harmful stimuli such as pathogens, damaged cells, toxic compounds, or irradiation [112]. Inflammation is characterized by redness, heat, swelling, and pain [113]. Critical microcirculatory events in inflammation involve changes in vascular permeability, the release of pro-inflammatory mediators, and the recruitment of leukocytes [114]. During acute inflammatory responses, cellular and molecular events reduce the risk of injury and lead to the resolution of inflammation. Nevertheless, uncontrolled acute inflammation can progress to chronic inflammation, which contributes to the onset of multiple chronic conditions [115].

An inflammation response elicits an increased influx of immune cells at the site of tissue injury. Initially, white blood cells roll along the vascular endothelial cells, a process mediated by selectins [116,117]. Upon activation by chemokines (such as CCL4, CX3CL1, and CXCL4) released from resident inflammatory cells, and presented in the luminal surface of the endothelium, leukocytes express integrins (like CD18), which bind to certain proteins (such as Intercellular Adhesion Molecule 1 or ICAM-1) [116,117]. This process enables leukocytes to migrate through the endothelium and reach the inflammatory sites.

**Figure 4:**
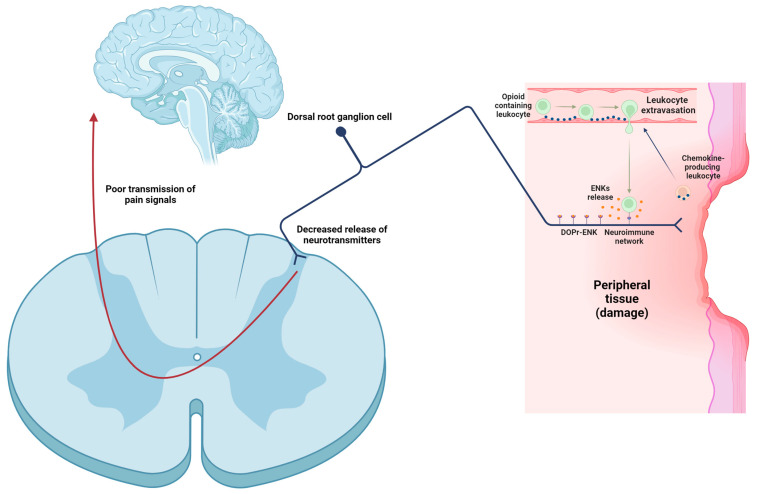
Involvement of leukocytes in peripheral pain control. Once peripheral tissue is damaged, resident leukocytes begin to release chemokines, which are presented on the luminal surface of endothelial cells. Opioid-containing leukocytes recognize these chemokines and cross the endothelial barrier. Subsequently, leukocytes interact with nociceptors and secrete ENKs. Upon release, ENKs bind to primary sensory fibers, thereby inhibiting the secretion of glutamate, CGRP, and substance P onto spinal neurons. This results in reduced transmission of pain signals to higher centers.

In inflammatory conditions, immune cells containing opioids (ENKs, among others) migrate to the inflamed tissue and secrete their content [118]. Several molecules can initiate the release of opioid peptides from immune cells, like noradrenaline (NA) [119], interleukin-1β (IL-1β) [120], CRF [120], CCL4 [121], and CXCL8 (IL-8) [122]. Next, opioid peptides pass through the damaged perineurial sheath and activate opioid receptors, located at the peripheral endings of primary afferent neurons, resulting in analgesia [87,88,89]. ENKs block the release of glutamate, substance P, and CGRP from these neurons [87,88,89], thereby reducing the transmission of pain signals to higher brain centers (Figure 4). This modulation of pain signaling is crucial for decreasing the intensity and perception of pain.

Peripheral pain control represents a breakthrough in the development of drugs that promote the release of ENKs [121] or replicate their effects [123] without needing to cross the blood–brain barrier (BBB) to achieve analgesic effects. If an opioid analgesic penetrates the BBB, such as morphine or fentanyl, it not only produces significant analgesic effects but also results in substantial side effects associated with opioid use, including tolerance and physical dependence [17].

2. Regulation of gastrointestinal function. Endogenous opioid peptides (including ENKs) have been localized to both enteric neurons and mucosal endocrine cells within the gastrointestinal tract [124]. In the ENS, derivatives of pENK are predominantly localized within myenteric neurons projecting to the circular muscle and submucosal plexus [124]. Upon secretion, ENKs increase the gastrointestinal transit time by interacting with DOPr on the enteric circuitries that control motility and secretion [125].

## 4. Relationship of Enkephalins with Painful Disorders

In previous sections, the mechanisms through which ENKs regulate pain intensity have been analyzed. However, this section will explore the role of ENKs in the regulation of pain associated with several diseases. Thereafter, the effectiveness of derivatives of ENKs in both preclinical and clinical studies will be described. The pathologies that will be analyzed are the following:

1. Fibromyalgia. Fibromyalgia is a chronic pain syndrome characterized by musculoskeletal pain accompanied by other non-specific symptoms [126]. Its prevalence in the United States is 6.4%, while in Europe and South America ranges from 2.4% to 3.3% [127,128,129]. Although the main cause of fibromyalgia remains unclear, it’s believed to involve a combination of many factors that involve genetics and/or environmental influences [130]. Symptoms of fibromyalgia differ among individuals but typically include pain (pain varies in intensity and location and is frequently accompanied by sensitivity in particular regions known as *tender points*, as defined by the American College of Rheumatology or ACR), fatigue (many people experience profound fatigue, even after sleeping for long periods), cognitive difficulties (issues with memory and concentration), frequent headaches, anxiety, depression, and sensitivity to noise or lights [130].

Several studies have confirmed a correlation between plasma/serum ENK levels and the severity of fibromyalgia. In preliminary research, the aim was to measure the plasma levels of Met-ENK following the topical application of lidocaine to tender points. The results showed a significant increase in Met-ENK levels, suggesting that this peptide might play a role in the analgesic effects of lidocaine [131]. Moreover, levels of opioid peptides, including MEAP, were assessed in the cerebrospinal fluid of patients with fibromyalgia. The results indicated higher concentrations of this opioid peptide, supporting previously published data [132]. Finally, a recent investigation quantified the activity of enkephalinases in patients with fibromyalgia. The results demonstrated that a small percentage of these patients had low enzyme activity [133]. Based on these results, it can be suggested that the organism activates numerous physiological mechanisms to relieve pain.

Although there have been limited studies conducted, investigating the role of ENKs in patients with fibromyalgia represents a promising research field that could result in the development of effective painkillers. In this regard, a recent publication [134] reveals that opiorphin, an opioid peptide discovered in the saliva of individuals [135], and its analogs (sialorphin, STR-324), evoke a strong inhibition of proteolytic enzymes that degrade ENKs, providing a promising alternative for the treatment of fibromyalgia. Conversely, there is a pharmacological intervention that involves low doses of naltrexone (non-selective opioid receptor antagonist; LDN therapy), which evokes the endogenous biosynthesis of Met-ENK [136]. This therapy is widely recognized for its efficacy in alleviating the pain associated with fibromyalgia [137,138].

2. Migraine. Migraine is a condition identified by either occasional headaches accompanied by symptoms like sensitivity to light (photophobia), or sound (phonophobia) [139]. This circumstance, that impacts on 15% of the global population [140], can also be linked to different symptoms such as somnambulism, emesis, abdominal migraine, benign paroxysmal positional vertigo (BPPV), benign paroxysmal torticollis (BPT), and confusional migraine, each characterized by singular clinical presentations [141]. A *migraine attack* can be divided into four phases based on its temporal sequence and symptoms: (i) *premonitory phase* (involves symptoms like mood changes or food cravings); (ii) *aura phase* (not always present; includes sensory or visual disturbances); (iii) *headache phase* (strong head pain, generally unilateral and pulsating); (iv) *postdrome phase* (leave individuals exhausted or disoriented, lasting for hours to days after the pain relief) [141]. These symptoms are caused by the activation of the trigeminovascular system, which triggers the release of certain vasoactive peptides, such as substance P and/or CGRP. This secretion leads to neurogenic inflammation and vasodilation of cerebral blood vessels [142].

In the 1980s, research into the modulation of pain by ENKs began. Plasma levels of Met-ENK were analyzed between control and migraine patients, and it was observed that amounts of Met-ENK in migraine patients were significantly higher [143,144,145,146,147]. Therefore, Met-ENK is considered a potential biomarker for migraine detection [143]. Two recent publications have demonstrated that inhibition of enkephalinase activity improves symptoms induced by migraines [148,149]. In this regard, researchers employed an animal model (migraine induced by i.p. administration of isosorbide dinitrate or ISDN) to investigate the analgesic effects of the Dual ENKephalinase Inhibitor (DENKI) PL37 [148]. This drug blocked the mechanical allodynia induced by ISDN [148]. In another rat model (migraine induced by i.p. injection of sodium nitroprusside or SNP), the administration of PL37 attenuated stress-induced facial hypersensitivity, and facial grimace responses [149].

Similarly to fibromyalgia, there have been limited studies assessing the effectiveness of ENKs (and derivatives) in managing migraines. However, recent research has indicated that DENKIs are excellent candidates for pain management [150]. Another interesting alternative could be the LDN therapy (in combination with dietary modifications), which has shown very promising results in a patient with migraine and multiple sclerosis [151]. Accordingly, it is convenient to continue investigating to find new effective treatments for migraine.

3. Complex Regional Pain Syndrome (CRPS). CRPS is a chronic pain characterized by allodynia and hyperalgesia, usually involving the limbs [152,153]. This term was proposed by the IASP in 1994, distinguishing it into type 1 (caused by injury) and type 2 (resulting from prior neurological damage) [152,153]. Signs and symptoms are disproportionate to the triggering event and include spontaneous or movement-induced pain, sensory changes (such as allodynia and hyperesthesia), autonomic dysfunctions (with many changes in biophysical properties of skin), and motor abnormalities (like tremors or dystonia) [152,153]. According to the European Medicine Agency (EMA) and the Food and Drug Administration (FDA), CRPS is designated as a rare disease, affecting fewer than 10,000 people in Europe or 200,000 individuals in the United States [154].

Recent studies have contributed to a deeper understanding of the pathophysiology of CRPS. The principal factor that contributes to CRPS is inflammation, which can occur in response to tissue damage [155]. TNF-α, IL-1β, and other pro-inflammatory cytokines are released throughout the inflammation process, inducing neuroinflammation and central sensitization [156]. This fact contributes to the development of chronic pain in CRPS patients [156]. Conversely, autonomic dysregulation is another factor behind CRPS, which result from nerve damage or inflammation [157]. Hence, in CRPS patients, autonomic dysfunction leads to changes in blood flow and skin temperature and symptoms like swelling and/or hypersensitivity to touch. Furthermore, other causes of CRPS can include brain and spinal cord changes in their connectivity, prompting neural alterations between different regions of the CNS [158].

In 2006, in a patient diagnosed with CRPS (along with other pathologies), increased plasma levels of Met-ENK were detected, indicating presumably an overactivation of the opioid system aimed to mitigate the adverse effects of this syndrome [159]. One possible effective intervention is the LDN therapy, which markedly reduces the activity of microglial cells [160,161]. Activation of these cells may be a vital factor in the onset and development of fibromyalgia and CRPS [162]. In summary, all these data represent a promising starting point for the search of novel ENK derivatives to combat the intense pain evoked by CRPS.

4. Rheumatoid arthritis. This disease, which affects 1% of the global population [163], is characterized by a chronic inflammatory process, which can lead to damage joints and extra-articular organs such as the heart, kidneys, lungs, gut, and brain [164]. Clinical symptoms of this pathology include morning stiffness, pain (shoulders, neck, and pelvic girdle), reduced mobility accompanied by fever, fatigue, weight loss, and the formation of rheumatoid nodules [164]. Rheumatoid arthritis is caused by crystal depositions (calcium pyrophosphate deposition disease or CPPD); by microbial agents (*S. aureus*, *N. gonorrhoeae*, several species of *Borrelia* -e.g., *B. burgdorferi*-, Parvovirus, and Enterovirus); or by autoimmune processes [164,165,166]. Risk factors for rheumatoid arthritis include age (older individuals show increased risk), sex (females exhibit higher incidence), smoke, obesity, and genetics [167].

The involvement of ENKs in rheumatoid arthritis-induced pain has been analyzed since the late 1980s. It began with the examination of various neuropeptides (including Met-ENK) in the synovial fluid of ill patients, and a higher amount of this opioid peptide has been detected [168,169,170]. Additionally, an increase in neutral endopeptidase (NEP) activity was observed in the synovial fluid of patients with rheumatoid arthritis, probably to compensate for the increase in quantities of Met-ENK [171]. These findings provide much evidence of the analgesic [169] and anti-inflammatory [172] properties of ENKs. According to these results, a viral vector (derived from herpes simplex virus type 1 or HSV-1), carrying the pENK cDNA, was designed to introduce it into sensory neurons and enhance the synthesis of ENKs. Promising results were achieved in rats with rheumatoid arthritis [173,174].

There is only one drug, not yet commercialized, designed to enhance the opioid system while suppressing the immune system activity. This drug, called enkorten, is composed by a combination of Met-ENK and tridecactide (alpha-corticotropin 1–13 or ACTH 1–13) [175]. Its toxic effects have been studied in rats and humans, with no issues reported [175]. Its efficacy against rheumatoid arthritis remains to be determined in both preclinical models and clinical trials, although it is presumed to be effective against rheumatoid arthritis and other pathologies (e.g., astma or multiple sclerosis) due to its anti-inflammatory, and analgesic effects [175].

To achieve analgesic (and anti-inflammatory) effects, it is essential to develop drugs that boost the opioid system.

5. Trigeminal neuralgia. According to the IASP, trigeminal neuralgia is a pathology identified by chronic facial pain [176,177,178]. Trigeminal neuralgia induces severe pain, defined by burning, sharp, and stabbing sensation [176,177,178]. Periods of pain are transient, with minor painful intervals between episodes [176,177,178].

This disease can be classified into three types, based on the International Headache Society (IHS) division [179]: (i) *classical trigeminal neuralgia* (the most prevalent type—75% of cases; the diagnosis is established when magnetic resonance imaging or MRI shows trigeminal neurovascular compression accompanied by morphological changes ipsilateral to the side of the pain); (ii) *secondary trigeminal neuralgia* (attributable to a specific identifiable neurological condition, with the exception of trigeminal neurovascular compression); (iii) *idiopathic trigeminal neuralgia* (diagnosed when no identifiable cause for trigeminal neuralgia can be determined).

Trigeminal neuralgia has an estimated prevalence of 0.3% [180], with an incidence rate of 4.5 per 100,000 individuals (and year), being more prevalent among women (ratio 3:2) [181]. This condition is much more common in the age group of 50 to 69 years [182]. Hypertension, arteriosclerotic vascular changes, aging, and familial history are the principal risk factors for trigeminal neuralgia [183]. Additionally, there is a higher incidence of trigeminal neuralgia reported in patients with hypertension compared with the healthy population [183]. Genetic transmission of this disease has been documented [176]. Finally, clinical observations have shown that emotional factors can also precipitate various forms of this neuralgia [183].

To date, there are only two studies that link the antihyperalgesic effects of ENKs in preclinical models. The first research, published in 2005 [184], demonstrated that the administration of HSV-1 containing the pENK cDNA directly into the trigeminal ganglion had antihyperalgesic effects in a trigeminal neuralgia rat model (induced by chronic constriction injury into the left infraorbital nerve) [184]. These results were confirmed in another trigeminal neuralgia rat model (originated from orofacial formalin administration) [185]. Conversely, in a rat model of trigeminal neuralgia (induced by chronic constriction of the infraorbital nerve), LDN therapy has relieved the pain associated with this neuralgia [186].

Currently, there are no known clinical trials employing ENKs (or their analogs) for alleviating this pain. This fact provides an excellent starting point for research in this area.

6. Multiple sclerosis. Multiple sclerosis is an autoimmune disorder that affects the CNS, where demyelination and axonal transection occur [187,188]. This pathology is defined by a destroyed myelin sheath, at different levels, of myelinated neurons in discrete regions through the CNS [187,188]. The most characteristic lesions are focal areas in the white matter of CNS, identified with MRI [189]. The classical presentations of multiple sclerosis comprise depression; diplopia; sexual dysfunction; unilateral optic neuritis; myelitis (impaired sensation, weakness, and ataxia); focal sensory disturbance (limb paresthesias); brainstem syndromes (intranuclear ophthalmoplegia, vertigo, hearing loss, and facial sensory disturbance); and fatigue [187,188,190]. In most subjects, multiple sclerosis initially manifests as episodes of reversible neurological deficits, frequently followed by a progressive deterioration in neurological function [187,188,190].

Multiple sclerosis is the most observed demyelinating pathology, displaying of different prevalence values, ranging from 100 per 100,000 habitants in North America and Europe to 2 per 100,000 individuals in Eastern Asia and sub-Saharan Africa [191]. Several risk factors contribute to the development of multiple sclerosis, including age, sex, vitamin D deficiency, genetic influences, smoke, injuries (into the CNS), and multiple infections (*Chlamydia*, and herpes simplex) [192]. Several theories have been proposed to explain the onset of multiple sclerosis, with two being the most widely accepted: *inside-out* (autoreactive CD4^+^ Th1 and Th17 cells destroy the myelin sheath) [192], and *outside-in* (initially, damage to the oligodendrocytes manifests, which subsequently triggers a strong inflammatory response) [193].

There is substantial evidence suggesting that endogenous opioid peptides (including ENKs) contribute to immune cells activity. Although the exact cause of multiple sclerosis remains nuclear, it is widely recognized that the immune system plays a role in the onset and progression of this pathology. Moreover, exploring the interaction between ENKs and the immune system in the context of multiple sclerosis is vital for understanding its pathophysiology.

Human and animal studies provide converging lines of evidence indicating that disruptions in the endogenous opioid system evokes the progression of this pathology. Mice with experimental autoimmune encephalomyelitis (EAE), the preclinical murine model that is most commonly used for research on multiple sclerosis, displayed a significant reduction in serum Met-ENK concentrations compared to controls prior to the onset of behavioral signs of this disease [194,195]. Furthermore, patients with multiple sclerosis displayed lower Met-ENK levels in peripheral blood cells and cerebrospinal fluid samples compared with healthy controls [194].

Given the role of Met-ENK in regulating the adaptive immune cell activity [196,197], diminished serum levels in multiple sclerosis patients may promote immune cell proliferation and reactivity. Indeed, several studies suggest that elevated serum levels of Met-ENK offer neuroprotective benefits in EAE mice, as well as in individuals with multiple sclerosis [194,198]. In the 1980s, one study demonstrated that recurrent injections of Met-ENK in EAE rats prevented or eliminated paralysis [199]. Repeated administration of Met-ENK to EAE mice inhibited disease progression and reduced the areas of demyelination and activated glial cells in the spinal cord, compared with the controls [195,199,200]. In EAE mice, treatment with Met-ENK stopped disease progression, improved clinical behavioral scores, and decreased the demyelinated areas in the spinal cord [201,202]. Moreover, treatment with Met-ENK in EAE mice resulted in a reduction in the severity of clinical disease scores [203].

Clinical studies on multiple sclerosis are difficult by several factors, such as the duration of the disease, the age of onset, and the reported symptoms. Furthermore, Met-ENK is not FDA-approved for clinical use in humans. Other treatments that are also appropriate for the management of pain include LDN therapy, which triggers several physiological mechanisms to boost the biosynthesis of Met-ENK [186]. In clinical studies, serum Met-ENK levels were measured following either disease modifying therapy or LDN leading to self-reported higher scores on the MS-QoL survey [204]. Through a clinical study (IRB protocol 9784), executed with volunteer patients at the Penn State Hershey Neurology Clinic, it was determined that serum Met-ENK levels were substantially lower in patients with multiple sclerosis compared with the control group [204]. However, due to the small sample size of the study and the demographic variability in disease duration and treatment length, reliable comparisons could not be made [205].

In conclusion, the results presented in this subsection demonstrate that modulating blood Met-ENK levels could be a significant target for treating pain related to multiple sclerosis.

7. Crohn’s disease. Crohn’s disease is an inflammatory bowel disease (IBD), typified by inflammation and ulceration of the digestive tract, being the most frequently affected part the distal ileum, but it can impact any portion of the intestine in a non-continuous manner [206,207]. The symptoms of this disease affect patients’ health and may comprise abdominal pain, diarrhea, weight loss, rectal bleeding, and fatigue [206,207]. In addition, Crohn’s disease can lead to severe complications such as intestinal obstruction, fistulas, and abscesses, which require hospitalization and surgery [206,207]. Crohn’s disease is more prevalent in the industrialized world, particularly in North America and Western Europe, although the incidence is rising in Asia and South America [208,209].

The pathogenesis of Crohn’s disease is complex and multifactorial, believed to entail complex interactions: (i) genetic factors (several genetic variants associated with Crohn’s disease have been identified, including genes related to T cell activity, and production of pro-inflammatory cytokines) [210]; (ii) environmental factors (diet, smoke, pollution, and exposure to specific bacteria and viruses increase the risk onset of Crohn’s disease) [211]; (iii) immunological factors (T lymphocytes, dendritic cells, and epithelial cells are believed to be involved in this pathology. Moreover, inflammatory cytokines such as TNF-α, IL-6, and IL-12 are thought to play crucial roles in the development of Crohn’s disease) [212].

In the 1980s, research was initiated to investigate the relationship between ENKs and Crohn’s disease. In a preliminary study with human samples, Leu-ENK fibers were located in the myenteric plexus of the ileum and colon. However, no quantitative differences were observed between both experimental groups (healthy vs. Crohn’s disease) [213]. A subsequent study observed that the Met-ENK content in humans with Crohn’s disease was significantly lower in the muscularis externa layer [214]. These data provided a starting point for investigating the association between ENKs and Crohn’s disease.

Another study (performed on humans) has demonstrated that although the density of Leu-ENK fibers is the same between groups, the vesicle content was significantly lower in the affected colonic samples [215]. These findings were correlated with another recent research, which determined the lower amounts of Met-ENK in the serum of patients with Crohn’s disease [216]. One possible explanation for the reduced levels of Met-ENK is an increase in dipeptidyl peptidase 4 (DPP-IV) activity in ill patients [217,218].

Based on these data, the next step involves searching for new drugs that effectively alleviate the pain caused by Crohn’s disease. As with the previously mentioned pathologies, LDN therapy has demonstrated efficacy in pain relief [219,220]. Therefore, it would be beneficial to develop new analogs of ENKs that also demonstrate antidiarrheal effects [221]. In this context, racecadotril (selective enkephalinase inhibitor) showed high effectiveness in treating acute diarrhea [222].

8. Cancer. Cancer develops as a result of multiple molecular events involving numerous interactions between genetics and environment [223]. This pathological process consists in a multistep phenomenon involving sequential mutations, resulting in uncontrolled cell growth [223]. These modifications alter the cellular metabolism, affect proliferation control, enable indefinite lifespan, alter intercellular communication, and provide the ability to avoid recognition by the immune cells [224]. Moreover, malignant cells become genetically damaged, but maintain their proliferative capacity [224].

In 2000, some researchers attempted to visualize the complexity of cellular changes found in cancer cells, which they termed *cancer hallmarks*. These hallmarks include: (i) persistent proliferative signaling; (ii) evading growth suppressors; (iii) avoiding apoptosis; (iv) unrestricted replicative potential; (v) promoting angiogenesis; (vi) stimulating invasion and metastasis; (vii) altered cellular metabolism; (viii) avoiding immune action; (ix) genomic instability and mutations; (x) tumor-promoting inflammation [225].

According to the Global Cancer Observatory (GLOBOCAN), there were an estimated 20.0 million new cancer cases worldwide in 2022 and 9.7 million deaths [226]. Lung cancer is the most commonly diagnosed cancer (12.4%), followed by female breast (11.6%), colorectal (9.6%), prostate (7.3%), and stomach (4.9%) cancers. Lung cancer is also the principal cause of cancer deaths (18.7%), followed by colorectal (9.3%), liver (7.8%), female breast (6.9%), and stomach (6.8%) cancers [226]. In a recent systematic review that included studies from 2014 to 2021, researchers discovered that the prevalence of pain among oncology patients was 44% [227]. Therefore, conducting new studies focused on controlling tumor development and related phenomena, such as inflammation and ischemia, could prove essential. Among the principal causes of cancer is tobacco consumption (cigarette smoking), alcohol abuse, obesity, lack of physical activity, infectious agents, sun exposure, and low fruit and vegetable intake [228].

Cancer pain can arise from tumor growth, which may compress surrounding tissues, or from metastasis to other parts of the body [229]. Furthermore, cancer treatments (such as surgery, chemotherapy, and radiotherapy) can either induce or exacerbate pain [229]. Effective management of cancer pain requires a multidisciplinary approach that includes pharmacological treatments (such as opioids, non-opioid analgesics, and adjuvant medications that improve the efficacy of analgesics) and non-pharmacological approaches (including physical and cognitive-behavioral therapies) [230]. Treating cancer pain is fundamental to enhance patients’ quality of life [231].

Opioid peptides (including ENKs) have been demonstrated to bind to their corresponding receptors, restraining tumor growth [232,233,234,235,236,237,238,239,240,241,242]. There are numerous studies that associate ENKs with tumor development: (i) control of tumor growth (commonly, Met-ENK exert inhibitory effects on tumor cells; however, certain tumor cell lines develop better in presence of this opioid peptide) [232,233,234,235,236,237,238,239]; (ii) immune modulation (Met-ENK repress the production of pro-inflammatory cytokines like TNF-α and IL-1β; additionally, ENKs are also implicated in the suppression of B and T lymphocytes) [196,239]; (iii) angiogenesis (Met-ENK inhibit the angiogenesis) [240]; (iv) metastasis (both Met-ENK and Leu-ENK negatively regulate this physiopathological process) [241,242]; (v) relief from cancer pain [243]. In conclusion, it is evident that opioid peptides demonstrate helpful effects in tumor development.

In many experimental models, including pancreatic [244], bone [243,245], and gastric [246] cancers, the administration of exogenous ENKs (or enkephalinase inhibitors, such as DENKIs), leads to a significant decrease in cancer pain intensity. In humans, the analgesic effects of ENK analogues [247], or ENK degradation inhibitors (tiorfan and bestatin) [248], have been documented. Furthermore, clinical trial No. NCT00109941 has clearly demonstrated that Met-ENK has antitumoral effects, as the median survival time for patients treated with Met-ENK was three times longer compared to that of untreated individuals with advanced pancreatic cancer [249].

According to these data, it can be concluded that enhancing the endogenous opioid system provides beneficial effects in managing cancer pain. However, additional research is essential to discover new compounds that enhance ENK function.

## 5. Conclusions

Although pain serves as a fundamental sensation to alert the body to potential health issues, it constitutes a highly unpleasant sensation. Currently, there are many drugs that control the intensity of pain through numerous physiological pathways, each with varying levels of efficacy. However, our organism has several mechanisms for regulating pain because we can synthesize ENKs, a subtype of opioid peptides that bind to DOPr.

Therefore, a comprehensive understanding of the analgesic mechanisms triggered by ENKs will facilitate the development of new painkillers, especially those that do not penetrate the BBB, thereby avoiding undesired opioid effects like tolerance and physical dependence. Despite the positive outcomes achieved with ENKs analogs, further research in this area is crucial.
